# An Enhanced Deep Learning Model for Effective Crop Pest and Disease Detection

**DOI:** 10.3390/jimaging10110279

**Published:** 2024-11-02

**Authors:** Yongqi Yuan, Jinhua Sun, Qian Zhang

**Affiliations:** 1 School of Information Technology, Jiangsu Open University, Nanjing 210000, China; yuanyq@jsou.edu.cn (Y.Y.); zhangqian@jsou.edu.cn (Q.Z.); 2 School of Design, Jiangsu Open University, Nanjing 210000, China

**Keywords:** crops, pest and disease recognition, image recognition, deep learning

## Abstract

Traditional machine learning methods struggle with plant pest and disease image recognition, particularly when dealing with small sample sizes, indistinct features, and numerous categories. This paper proposes an improved ResNet34 model (ESA-ResNet34) for crop pest and disease detection. The model employs ResNet34 as its backbone and introduces an efficient spatial attention mechanism (effective spatial attention, ESA) to focus on key regions of the images. By replacing the standard convolutions in ResNet34 with depthwise separable convolutions, the model reduces its parameter count by 85.37% and its computational load by 84.51%. Additionally, Dropout is used to mitigate overfitting, and data augmentation techniques such as center cropping and horizontal flipping are employed to enhance the model’s robustness. The experimental results show that the improved algorithm achieves an accuracy, precision, and F1 score of 87.09%, 87.14%, and 86.91%, respectively, outperforming several benchmark models (including AlexNet, VGG16, MobileNet, DenseNet, and various ResNet variants). These findings demonstrate that the proposed ESA-ResNet34 model significantly enhances crop pest and disease detection.

## 1. Introduction

Plant pests and diseases are an ongoing cause of potentially severe impacts on crop growth and yield [1]. Outbreaks of pests and diseases can lead to reduced crop production, decreased quality, and even complete crop failure, posing threats to food security and directly affecting farmers’ livelihoods and national economic stability [2]. Early detection of crop pests and diseases is fundamental for effective prevention and control, playing a crucial role in agricultural production management and decision-making [3]. Therefore, researching and addressing plant pest and disease issues is of utmost importance.

With the advancement of agricultural modernization, traditional pest and disease control methods can no longer meet the demands of large-scale agricultural production. These conventional methods are not only time-consuming and labor-intensive, but also require extensive professional knowledge and experience [4]. As such, improving the efficiency and accuracy of pest and disease detection through advanced technological means has become a research hotspot.

Diseased crops exhibit noticeable lesions or markings on their leaves, stems, flowers, and fruits, with each pest or disease having unique characteristics. Traditional plant pest and disease image recognition methods typically rely on manually designed feature extractors and machine learning algorithms [5,6]. These methods require careful selection and construction of features, such as texture, color, and shape, followed by image segmentation to achieve classification [7]. While experienced agricultural experts can identify pests and diseases through visual inspection or microscopy, this approach is often time-consuming, labor-intensive, and subject to human bias. Moreover, different types of crops and pests necessitate continuous adjustment and optimization of feature extractors, increasing system complexity and maintenance costs. In real-world natural environments, plant pest and disease detection faces numerous challenges, such as the low contrast between diseased areas and the background, varying scales and types of diseased areas, and interference when images are captured under natural lighting conditions [8]. Traditional methods often fail to achieve satisfactory detection results under these circumstances [6,9].

In recent years, deep learning technology has rapidly advanced image segmentation, classification, and object detection, providing new possibilities for plant pest and disease image recognition. Deep learning models, especially convolutional neural networks (CNNs), can automatically learn features from images without manual design, making the automatic identification of plant pests and diseases more efficient and accurate. Furthermore, deep learning can handle various types of crops and pests, demonstrating better generalization capabilities. As a result, deep learning-based methods for plant pest and disease image recognition have become a hot research topic. These image recognition technologies have shown tremendous potential in agriculture, particularly in monitoring and identifying crop pests and diseases [10]. Various AI tools have made significant strides in pest control, and large-scale datasets contribute to the development of smarter and more efficient AI tools [11]. The healthy development of agriculture increasingly relies on the assistance of AI tools, enabling significant improvements in crop yields and promoting sustainable agricultural practices.

Deep learning-based image recognition technology has demonstrated great potential in agriculture, especially in monitoring and recognizing crop pests and diseases. These technologies can significantly improve crop yields and promote sustainable agricultural practices. For instance, models such as LeNet5 [12], AlexNet [13], VGGNet [14], GoogLeNet [15], InceptionV3 [16], ResNet [17], and DenseNet [18] have greatly enhanced the accuracy of plant disease recognition.

Despite the significant progress made by researchers in crop pest and disease image recognition, several challenges remain [19]. These include low model accuracy, poor performance in multi-class pest and disease classification, and the limited availability of existing agricultural pest and disease datasets [20]. Developing stable training models from these limited datasets is difficult. Additionally, the complexity of existing network models poses challenges, especially in agricultural regions with poor network connectivity, where there is a need to deploy suitable lightweight models on limited mobile devices [21]. To address these issues, this paper proposes an improved crop pest and disease image recognition algorithm based on ResNet34. The algorithm introduces an efficient spatial attention mechanism to better capture key information in images. Additionally, depthwise separable convolutions are used to replace traditional convolutions, reducing the model’s parameter count and computational complexity, thereby achieving a lightweight model. To mitigate overfitting, Dropout regularization is introduced [22], and data augmentation techniques such as center cropping and random horizontal flipping are employed to enhance model robustness. Extensive experiments on the AI Challenger 2018 public dataset validate the effectiveness of the proposed method. The contributions of this paper are summarized as follows:

1. An efficient spatial attention module (effective spatial attention, ESA) is proposed to improve the ResNet34 model, enabling the network to focus on key regions of images and extract critical features for better classification performance.

2. Depthwise separable convolutions are utilized to replace the standard 3×3 convolutions in the ResNet34 model, significantly reducing the model’s parameters and computational load, resulting in a more lightweight model.

3. Extensive experiments on the AI Challenger 2018 public dataset validate the effectiveness of the proposed method, demonstrating significant improvements in classification performance.

## 2. Related Works

Accurately identifying plant diseases is crucial for implementing timely control measures, which are essential for maintaining agricultural productivity and food security. Traditional disease diagnosis methods typically rely on expert knowledge and manual inspection, which are prone to human error and are insufficient to meet the needs of modern agriculture. The advent of digital image processing and recent advances in deep learning have revolutionized this field by enabling automated and highly accurate detection systems.

Deep learning, particularly convolutional neural networks (CNNs), has become the cornerstone for computer vision tasks, including plant disease recognition. CNNs excel at capturing the spatial hierarchies in images, making them well suited for pattern recognition in complex visual data, such as diseased plant leaves [23]. The foundational work by Krizhevsky et al. [13] with AlexNet demonstrated the potential of deep learning for visual recognition, paving the way for its application in plant pathology.

Pioneering work in agricultural pest detection used CNNs to develop an automated system for crop disease identification. Mohanty et al. [3] implemented a deep learning model to classify 26 diseases across 14 crop species with high accuracy, showcasing the potential of CNNs in agricultural applications. Similarly, Sladojevic et al. [24] used deep CNNs to identify 13 different types of plant diseases from leaf images, achieving an accuracy of 96.3%. Ferentinos et al. [25] made further progress by comparing various CNN architectures, including AlexNet, VGG, and GoogLeNet, for plant disease detection. Their study highlighted the effectiveness of deep learning models in handling complex image data and achieving high classification performance. Some studies have also explored using transfer learning to enhance model accuracy and reduce training time, showing promising results in pest detection. Lin et al. [26] proposed an adaptive feature fusion pyramid network for multi-class agricultural pest detection, extracting richer pest features and constructing a two-stage region-based CNN (R-CNN) to refine prediction bounding boxes, thereby determining the category and location of pests in each image. Qian et al. introduced a novel automated data augmentation method that adaptively searches for suitable augmentation strategies, modeling the data more effectively. They also employed Res2Net as the backbone to capture more detailed pest information and incorporated a reverse feature fusion layer into the feature pyramid network (FPN) to learn more details.

Attention mechanisms can enhance deep learning models by focusing on important regions of input images. Wang et al. [27] proposed a residual attention network for image classification, integrating attention modules into the residual blocks of ResNet. This approach significantly improved the model’s ability to capture relevant features and increased classification accuracy. Yuan et al. [28] proposed an attention module for image segmentation called TBSFF. By utilizing multi-scale context fusion and a feature selection network, it extracts richer semantic features and detailed information to achieve improved segmentation performance. In agricultural pest detection, attention mechanisms have been used to enhance model performance. Chen et al. [29] incorporated channel attention mechanisms into CNNs for plant disease recognition, achieving superior performance compared to standard CNN models. Similarly, of attention mechanisms in improving detection accuracy and reducing false positives. Liu et al. [30] introduced multi-scale convolutional kernels and coordinate attention mechanisms in SqueezeNext to enhance the model’s ability to extract lesions.

The demand for lightweight models deployable on resource-constrained devices has led to the exploration of depthwise separable convolutions. Howard et al. [31] introduced the MobileNet architecture which uses depthwise separable convolutions to significantly reduce parameter count and computational complexity, making it suitable for mobile and embedded applications. This approach has been used in various agricultural image analysis studies, enabling the development of efficient models deployable in real-world scenarios.

Evaluating deep learning models on large agricultural datasets is crucial for validating their effectiveness. The AI Challenger 2018 dataset has been widely used in recent research for benchmarking pest and disease detection algorithms. Gao et al. [32] evaluated their proposed dual-branch efficient channel attention-based crop pest and disease recognition model using this dataset. They noted that the AI Challenger 2018 Crop Disease Detection dataset presents a high level of difficulty, making it well suited for assessing model performance. Liu et al. [30] modified the categories in the AI Challenger 2018 dataset and augmented the dataset with external data. Following these changes, they restructured the training and validation sets and evaluated the proposed lightweight convolutional network.

Despite significant progress in automatic plant disease recognition, challenges such as low accuracy, poor multi-class classification performance, and model complexity persist [33]. These challenges are particularly evident when models are deployed in real-world agricultural environments where precision is critical. Our work addresses these challenges head-on by introducing an innovative algorithm leveraging the power of ResNet34, a robust architecture known for its effectiveness in handling complex image recognition tasks. However, we go beyond utilizing an existing framework by integrating an effective spatial attention mechanism. This new module, termed effective spatial attention (ESA), aims to enhance the model’s focus on key image regions, extracting critical features essential for accurate classification.

Incorporating depthwise separable convolutions into our model is another step toward addressing complexity issues. By replacing traditional convolutions with this more efficient alternative, we significantly reduce the model’s parameter count and computational load, resulting in a lightweight yet powerful solution. This approach aligns with the current trend of developing models that are not only accurate but also deployable in resource-limited environments [34].

To combat overfitting and enhance the model’s generalization ability, we introduced Dropout as a regularization technique [22]. This strategic addition ensures our model maintains high performance without overfitting the training data. Additionally, we enhanced the model’s robustness through data augmentation techniques, including center cropping and random horizontal flipping, which simulate various real-world conditions and help improve model resilience.

We rigorously validated the proposed method on the AI Challenger 2018 public dataset, demonstrating its effectiveness. This dataset, known for its diversity and complexity, provides a stringent benchmark for assessing our algorithm’s performance. The significant improvements we achieved in classification performance underscore the practical value and potential impact of our work.

In conclusion, our research contributes to the field not only by addressing existing challenges but also by introducing innovative solutions that push the boundaries of current technology. The integration of the ESA module, the adoption of depthwise separable convolutions, and comprehensive experimental validation collectively represent significant advancements in plant pest and disease image recognition.

## 3. Materials and Methods

Based on the above analysis, we recognize that while ResNet is sufficient for pest and disease identification tasks, its performance is limited by its inability to focus on key areas of the images and its relatively large model size, which is unsuitable for deployment on mobile devices. Therefore, we propose the ESA-ResNet34 network, an improved version of ResNet34, specifically designed for crop pest and disease detection. This network integrates an effective spatial attention (ESA) mechanism and depthwise separable convolutions to enhance the network’s focus on important regions of the input images, thereby improving performance while reducing computational complexity, making it suitable for deployment on mobile devices.

### 3.1. Network Architecture

**Figure 1:**
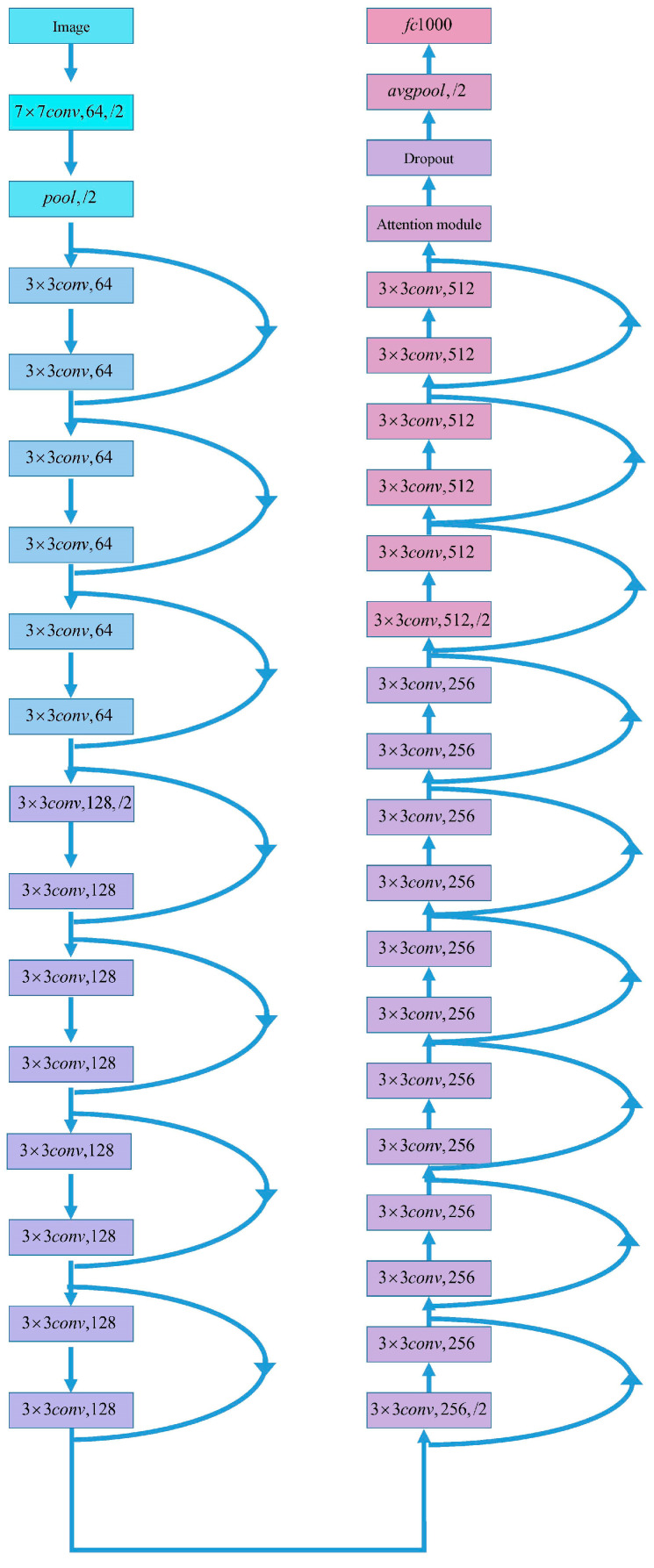
ESA-ResNet34 network structure.

Figure 1 presents our designed ESA-ResNet34 network. The ESA-ResNet34 network is built upon the ResNet34 architecture, known for its deep residual learning framework. ResNet34 employs skip connections to mitigate the vanishing gradient problem, enabling the training of deeper networks [17]. Our modifications to the ResNet34 architecture include the following:

1. Effective spatial attention (ESA) mechanism: We introduce the ESA mechanism to the network to improve its focus on important regions of the input images. This module enhances the model’s ability to capture critical features necessary for accurate classification.

2. Depthwise separable convolution: To reduce the number of parameters and the computational load of the model, we replaced standard convolutions with depthwise separable convolutions. This change significantly decreases the complexity of the model.

### 3.2. Effective Spatial Attention

Attention mechanisms are widely used in deep learning, simulating human perception and attention processes to allow neural networks to selectively focus on different parts of input data, thereby improving model performance. Significant breakthroughs have been achieved with attention mechanisms in both natural language processing and computer vision [35,36].

**Figure 2:**
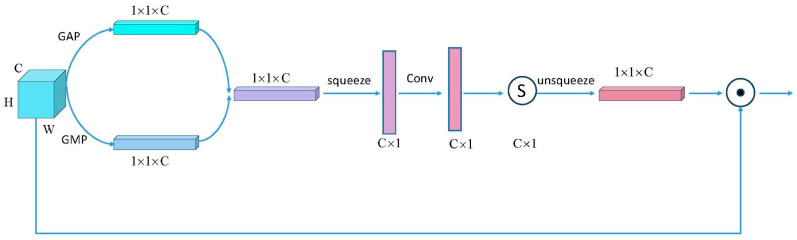
Efficient spatial attention mechanism.

The effective spatial attention (ESA) module proposed in this paper, illustrated in Figure 2, is an attention mechanism for convolutional neural networks that avoids dimensionality reduction to minimize the impact on network performance while capturing cross-channel interactions efficiently.

As shown in Figure 2, the input feature map first passes through a pooling layer to obtain a pooled 1×1×C feature map. Next, dimension swapping and vector dimensionality reduction are applied to create a transformed C×1 feature map. A 1×1 convolution operation is then used to efficiently extract features from this transformed map, followed by Sigmoid activation. Finally, by reversing the dimension swap and expanding the vector dimensions, the 1×1×C feature map is reconstructed. This reconstructed feature map is then multiplied with the original input feature map to apply the attention mechanism.

The proposed attention mechanism module does not require dimensionality reduction of the feature map channels, thus eliminating the need to input the number of feature map channels into the attention mechanism beforehand. This allows for adaptive attention to feature channels. The spatial attention map generated from the input feature map highlights key regions of the image relevant for classification. The spatial attention map is then applied to the input feature map, emphasizing important regions and suppressing irrelevant ones. This process enables the network to focus on the most informative parts of the image.

In summary, the ESA module improves model performance by allowing it to concentrate on crucial areas of the input image without the need for dimensionality reduction, making it more efficient and effective for image classification tasks in plant disease and pest detection.

### 3.3. Depthwise Separable Convolutions

**Figure 3:**
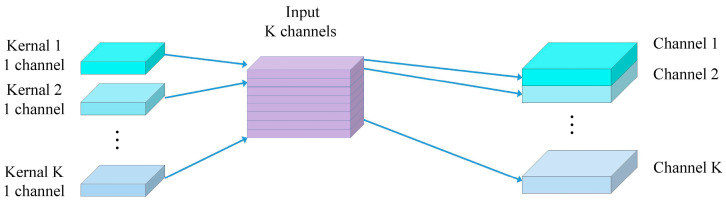
Depth convolution.

**Figure 4:**
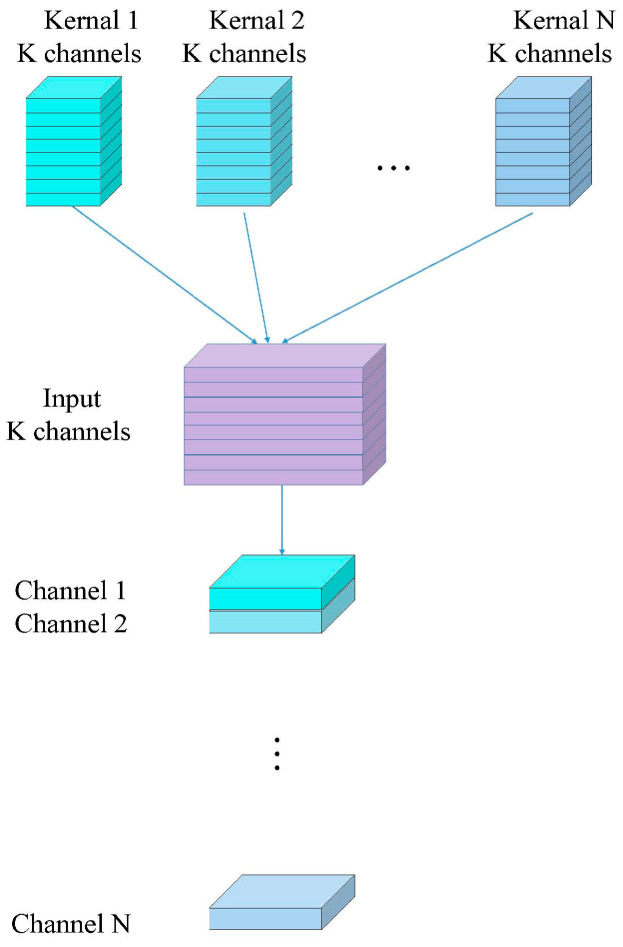
Standard convolution.

Depthwise separable convolutions consist of two main parts: depthwise convolutions and pointwise convolutions [37]. Depthwise convolution is computationally simple. As illustrated in Figure 3, it involves applying a convolutional filter to each input channel separately and then concatenating the outputs from all filters to obtain the final output of the depthwise convolution. The number of output channels is equal to the number of filters used. Since depthwise convolution applies one filter per channel, the number of output channels for each individual channel is also one. For example, if the input feature map has the dimensions (4, 64, 224, 224), applying one filter per channel results in 64 feature maps with one channel each. These 64 feature maps are then concatenated to produce an output feature map with 64 channels. Depthwise convolution is shown in Figure 3, while standard convolution is depicted in Figure 4.

**Figure 5:**
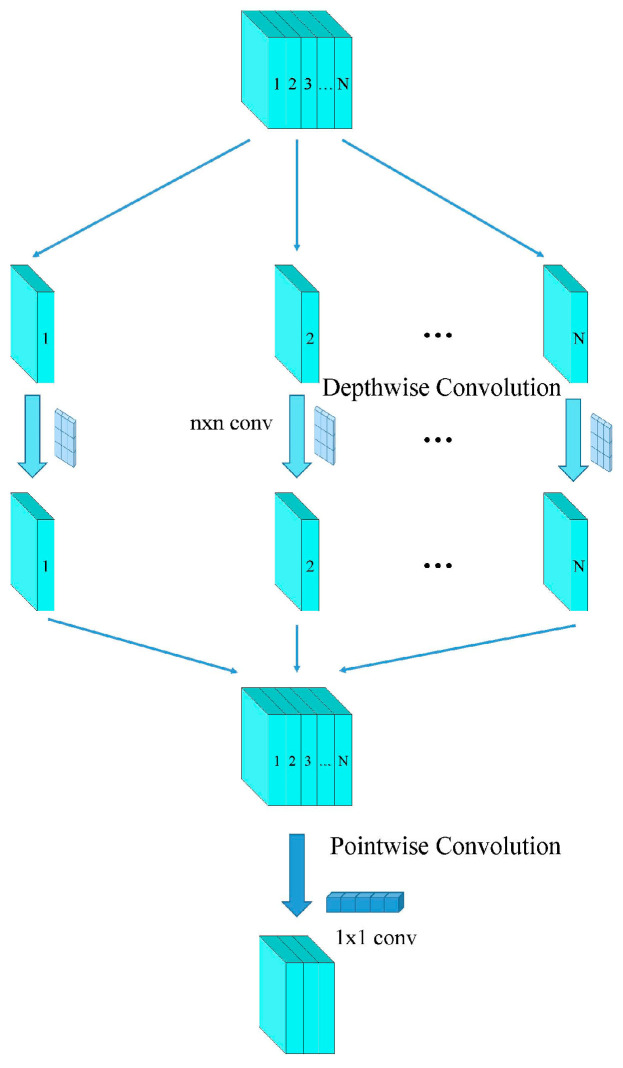
Depthwise separable convolution.

Pointwise convolution, essentially a 1×1 convolution, serves to adjust the number of output channels and to fuse the features extracted by depthwise convolution. Depthwise convolution alone cannot alter the number of input channels; it simply applies convolution to each channel independently and concatenates the results. Thus, if the input feature map has 64 channels, the output feature map after depthwise convolution will also have 64 channels. Pointwise convolution is then used to combine these features, enabling interactions between different channels and adjusting the number of output channels as needed. Pointwise convolution, as shown in Figure 5.

To reduce the complexity of the model, we replaced the 3×3 convolutions in ResNet34 with depthwise separable convolutions. In the original ResNet34, 3×3 convolutions are used to adjust the feature map to the required size and to modify the number of channels. In our approach, we utilize depthwise and pointwise convolutions. 

First, we apply a 3×3 depthwise convolution to the input feature map, which adjusts the spatial dimensions of the feature map without changing the number of channels. Then, a 1×1 pointwise convolution is used to adjust the number of channels without altering the spatial dimensions of the feature map. This approach significantly reduces the computational load of convolution operations, thereby lowering the overall complexity of the model.

## 4. Experiment

### 4.1. Implementation Details

The experiments were conducted using the PyTorch framework for both training and testing. The training and testing were performed on the Jiutian Bisheng platform with the following hardware configuration: an Intel(R) Xeon(R) Gold 6240 CPU @ 2.60 GHz, 32 GB of RAM, and an NVIDIA V100 GPU with 32 GB of VRAM. The software environment included CUDA Toolkit 11.1, Python 3.9.7, PyTorch 1.8.0, torchvision 0.9.0, and the Linux operating system.

In the experiments, we compared our improved model with AlexNet, VGG16, MobileNet, DenseNet, and other branches of ResNet. To ensure the reliability of the experimental results, all comparison models were implemented using the official models provided in Torchvision. Additionally, to facilitate the reproducibility of the results reported in this paper, we fixed the random seed to 2022. The training parameters utilized the Adam optimization method, with a weight decay of 1 × 10^−4^, a learning rate of 0.001, and the learning rate decay mode set to minimum mode. The learning rate reduction factor was set to 0.5, the batch size was 128, and the number of epochs was 200. The model was terminated early if there was no improvement in accuracy after 50 consecutive epochs. The loss function used was the cross-entropy loss, calculated as follows:

(1) $$L=\frac{1}{N}\sum_{i}L_{i}=-\frac{1}{N}\sum_{i}\sum_{c=1}^{M}y_{ic}\log\left(p_{ic}\right)$$

where M is the number of categories for classification. $y_{\mathrm{ic}}$ is an indicator function that takes the value 1 if the class of image i is c; otherwise, it is 0. $p_{ic}$ is the predicted probability that image i belongs to category  c.

### 4.2. Dataset

**Figure 6:**
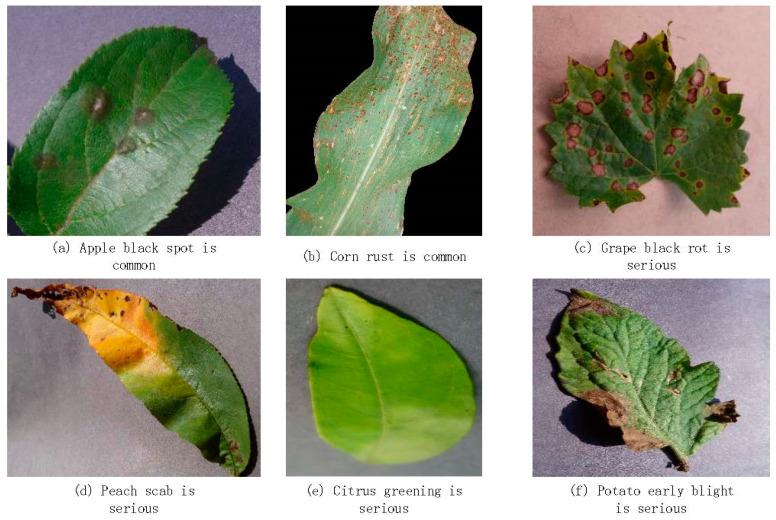
Crop pest and disease image examples.

The dataset used in this study is the AI Challenger 2018 pest and disease detection dataset. This dataset comprises a total of 50,000 annotated images, covering 61 diseases across 10 types of plants. We used the training set from AI Challenger as our training data and the validation set from AI Challenger as our test data. Example samples from the experimental dataset are shown in Figure 6.

### 4.3. Evaluation Metrics

To evaluate the proposed model, we used accuracy, precision, and F1 score as the performance metrics.

(2) $$\;\mathrm{Accuracy}=\frac{TP+TN}{TP+TN+FP+FN}$$

(3) $$\;\mathrm{Precision}=\frac{TP}{TP+FP}$$

(4)  F1 Score=2⋅Precision⋅RecallPrecision+Recall

*TP* (True Positive): the number of samples correctly predicted as positive by the model.

*TN* (True Negative): the number of samples correctly predicted as negative by the model.

*FP* (False Positive): the number of samples incorrectly predicted as positive by the model.

*FN* (False Negative): the number of samples incorrectly predicted as negative by the model.

### 4.4. Experimental Results

**Figure 7:**
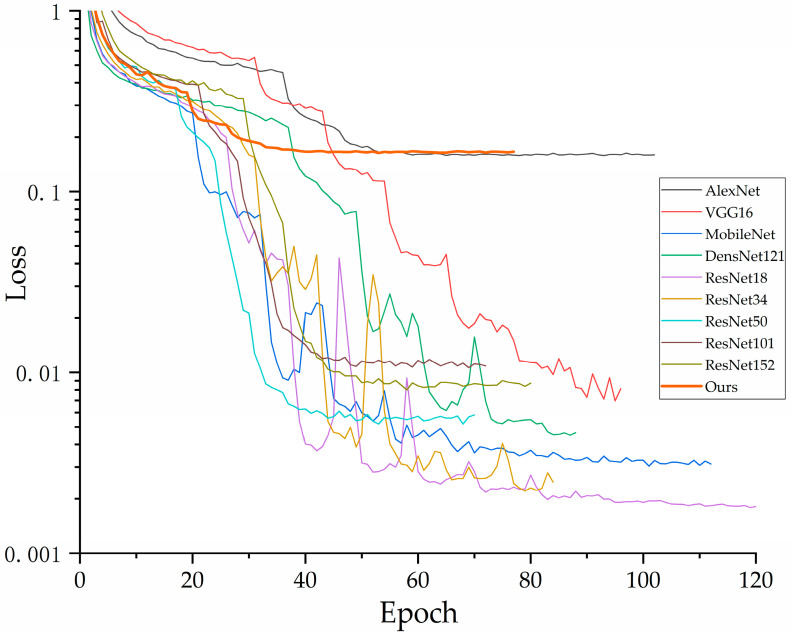
Training loss decline curve of each model.

**Figure 8:**
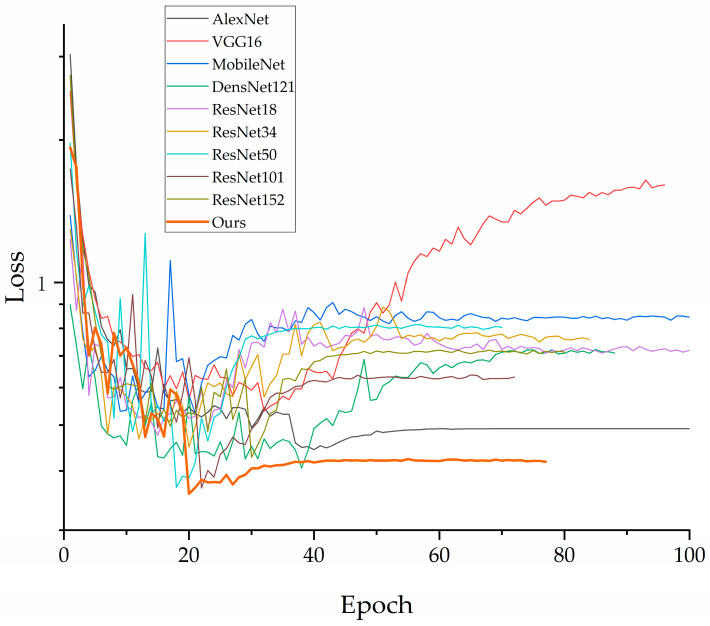
Loss decline curve of each model on validation set.

The accuracy and loss values for the training and validation sets of each model are shown in the figures below. As illustrated in Figure 7, the VGG16 model exhibits fluctuations in loss as it decreases, which is partially attributed to the class imbalance present in the dataset used in this study. In contrast, other models exhibit a gradual convergence as the loss decreases. Figure 8 indicates an upward trend in the validation loss curve for VGG16, which supports the previous analysis. 

**Figure 9:**
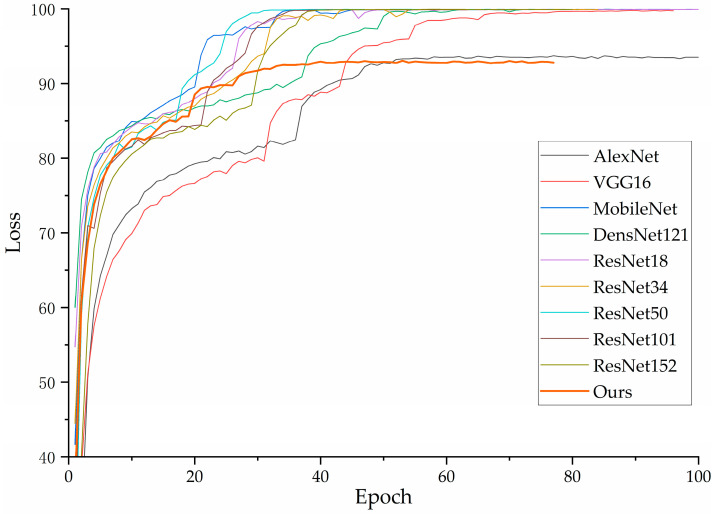
Accuracy rising curve of each model on training set.

**Figure 10:**
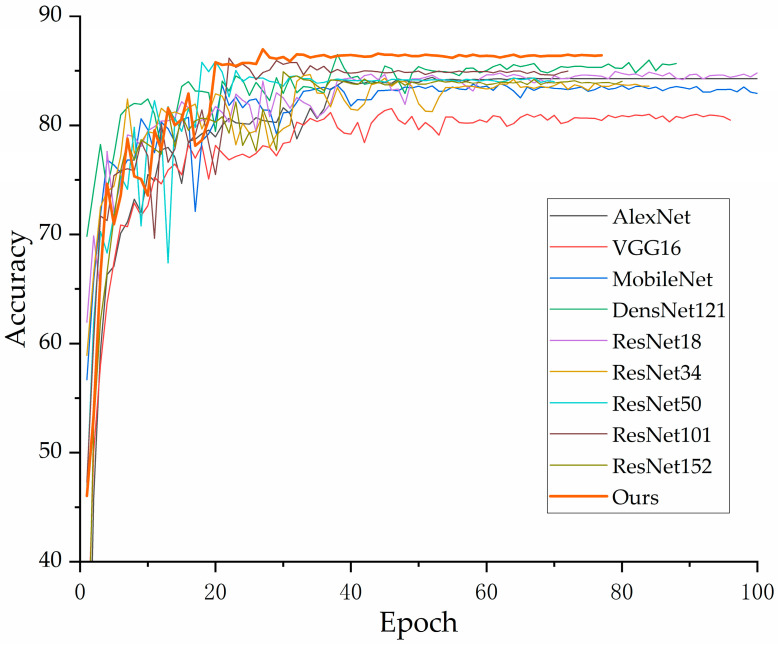
Accuracy rising curve of each model on validation set.

In Figure 9, it can be observed that the accuracy of each model converges to a fixed value, indicating that all models have achieved their maximum accuracy. Figure 10 shows that our proposed model delivers the best performance on the validation set, confirming its superior effectiveness. Additionally, Figure 7 demonstrates that all models ceased training before reaching 200 epochs. Figure 9 further indicates that all models achieved their best performance before 200 epochs, highlighting the importance of the early stopping mechanism employed in this study, which helps in reducing experimental resource wastage.

As shown in Table 1, the improved model proposed in this study achieved performance metrics of 87.09% accuracy, 87.14% precision, and 86.91% F1 score on the AI Challenger 2018 pest and disease detection dataset. These results significantly surpass those of other models, demonstrating the superiority of our model in pest and disease detection methods.

### 4.5. Ablation Study

To validate the effectiveness of the proposed improvements, we conducted ablation experiments on the proposed model. In machine learning, an ablation study refers to the process of systematically removing or modifying specific components or techniques of a model to evaluate their individual contribution to the model’s overall performance. The results are presented in Table 2. As shown in Table 2, the accuracy, precision, and F1 score of the ResNet34 model with the proposed attention module improved from 83.83%, 84.12%, and 83.75% to 85.77%, 85.92%, and 85.67%, respectively. This demonstrates the effectiveness of the proposed attention mechanism. Additionally, after replacing standard convolutions with depthwise separable convolutions, there was no significant decline in the model’s performance metrics, indicating that depthwise separable convolutions have minimal impact on the proposed model. Finally, the performance improvement observed with Dropout and data augmentation suggests that these techniques can enhance the model’s generalization ability to some extent, highlighting the importance of Dropout and data augmentation in computer vision tasks.

### 4.6. Model Complexity Evaluation

To evaluate and compare model complexity, we used the ‘thop’ library to assess both the proposed model and the comparison models. The evaluation was conducted assuming an input size of (*1*, *3*, *224*, *224*). The number of parameters and floating-point operations (FLOPs) for each model are presented in Table 3. From the data in Table 3, it is evident that the proposed improved model demonstrates significant advantages over ResNet34. Specifically, the proposed model successfully reduces the number of parameters by 85.37% and the number of FLOPs by 84.51% compared to ResNet34. This result strongly supports the efficiency of using depthwise separable convolutions to reduce model complexity.

Further analysis of Table 3 reveals that the proposed model not only outperforms other ResNet variants in terms of model complexity but also significantly reduces complexity compared to well-known models such as AlexNet, VGG16, and DenseNet. This indicates that the proposed model achieves a good balance between performance and efficiency, delivering better performance with minimal model complexity. Overall, this study effectively reduces the complexity of deep learning models while achieving enhanced performance, which is crucial for improving the efficiency and applicability of deep learning models.

## 5. Conclusions

This paper proposes an efficient spatial attention (ESA) module that effectively focuses on key features of images, enhancing recognition performance. The proposed ESA-ResNet34 model demonstrated exceptional results on the AI Challenger 2018 pest detection dataset, achieving results of 87.09, 87.14, and 86.91 for accuracy, precision, and F1 score, respectively. These results significantly surpass those of classical models such as AlexNet, VGG16, MobileNet, and DenseNet, indicating the model’s effectiveness in crop pest identification.

Furthermore, by replacing the standard convolutions in ResNet34 with depthwise and pointwise convolutions through the use of depthwise separable convolutions, the modified model successfully reduced the number of parameters by 85.37% and the floating-point operations by 84.51%. This reduction enhances model efficiency and supports its potential deployment on mobile devices, demonstrating that even with a significant decrease in model complexity, its performance has still improved. Additionally, the use of Dropout regularization and data augmentation methods in this study contributes to enhancing the model’s generalization ability and overall performance.

However, this paper also has certain limitations. Further research is needed to enhance the model’s robustness and generalization, and improvements are required to address the dataset imbalance. Additionally, the dataset used in this study was curated and cleaned by the data creators, but images of pests and diseases in real-world environments can be more complex. These images may suffer from issues such as blurriness, raindrop interference, or dust obstruction. In the future, it would be advisable to consider data augmentation techniques on the images before proceeding with pest and disease identification.

## Figures and Tables

**Table 1:** Performance of each model.

Model	Accuracy	Precision	F1 Score
AlexNet	83.28	83.13	83.06
VGG16	80.79	84.24	81.89
MobileNet	83.46	83.51	83.32
Densenet121	85.15	85.50	85.00
ResNet18	83.94	83.75	83.72
ResNet34	83.83	84.12	83.75
ResNet50	85.42	85.88	85.33
ResNet101	86.10	86.34	85.91
ResNet152	85.15	85.27	85.11
DECA_ResNet18 [32]	86.35	84.15	83.60
Ours	87.09	87.14	86.91

**Table 2:** Performance of each model in ablation experiment.

Model	Accuracy	Precision	F1 Score
ResNet34	83.83	84.12	83.75
ResNet34 + ESA	85.77	85.92	85.67
ResNet34 + ESA + Depthwise	85.48	86.06	84.22
Ours (ResNet34 + ESA + Depthwise + Dropout)	87.09	87.14	86.91

**Table 3:** Model complexity comparison.

Model	Params	Flops
VGG16	134.51 M	15.48 GFlops
AlexNet	57.25 M	0.71 GFlops
Densenet	7.02 M	2.90 GFlops
ResNet18	11.21 M	1.82 GFlops
ResNet34	21.32 M	3.68 GFlops
ResNet50	23.63 M	4.13 GFlops
ResNet101	42.63 M	7.86 GFlops
ResNet152	58.27 M	11.60 GFlops
Ours	3.12 M	0.57 GFlops

## Data Availability

The dataset used in this paper can be downloaded via this link: https://aistudio.baidu.com/datasetdetail/76075 (accessed on 29 October 2024).
